# Laparoscopic surgery for gallstones or common bile duct stones: A stably safe and feasible surgical strategy for patients with a history of upper abdominal surgery

**DOI:** 10.3389/fsurg.2022.991684

**Published:** 2022-09-30

**Authors:** Shaojie Yang, Shuodong Wu, Wanlin Dai, Liwei Pang, Yaofeng Xie, Tengqi Ren, Xiaolin Zhang, Shiyuan Bi, Yuting Zheng, Jingnan Wang, Yang Sun, Zhuyuan Zheng, Jing Kong

**Affiliations:** ^1^ Biliary Surgery (2nd General) Unit, Department of General Surgery, Shengjing Hospital of China Medical University, Shenyang, China; ^2^ Innovation Institute of China Medical University, Shenyang, China; ^3^ Breast Surgery Unit, Department of General Surgery, The Second Affiliated Hospital Zhejiang University School of Medicine, Hangzhou, China; ^4^ Department of Cardiology, Shengjing Hospital of China Medical University, Shenyang, China; ^5^ Department of Urinary Surgery, Taizhou Enze Medical Center (Group) Enze Hospital, Taizhou, China

**Keywords:** laparoscopic cholecystectomy, laparoscopic common bile duct exploration, previous upper abdominal surgery, abdominal adhesion, conversion to open surgery

## Abstract

**Backgrounds/Aims:**

A history of upper abdominal surgery has been identified as a relative contraindication for laparoscopy. This study aimed to compare the clinical efficacy and safety of laparoscopic cholecystectomy (LC) and laparoscopic common bile duct exploration (LCBDE) in patients with and without previous upper abdominal surgery.

**Methods:**

In total, 131 patients with previous upper abdominal surgery and 64 without upper abdominal surgery underwent LC or LCBDE between September 2017 and September 2021 at the Shengjing Hospital of China Medical University. Patients with previous upper abdominal surgery were divided into four groups: group A included patients with previous right upper abdominal surgery who underwent LC (*n* = 17), group B included patients with previous other upper abdominal surgery who underwent LC (*n* = 66), group C included patients with previous right upper abdominal surgery who underwent LCBDE (*n* = 30), and group D included patients with previous other upper abdominal surgery who underwent LCBDE (*n* = 18). Patient demographics and perioperative outcomes were retrospectively analyzed.

**Results:**

The preoperative liver function indexes showed no significant difference between the observation and control groups. For patients who underwent LC, groups A and B had more abdominal adhesions than the control group. One case was converted to open surgery in each of groups A and B. There was no statistical difference in operation time, estimated blood loss, postoperative hospital stay, and drainage volume. For patients who underwent LCBDE, groups C and D had more estimated blood loss than the control group (group C, 41.33 ± 50.84 vs. 18.97 ± 13.12 ml, *p* = 0.026; group D, 66.11 ± 87.46 vs. 18.97 ± 13.12 ml, *p* = 0.036). Compared with the control group, group C exhibited longer operative time (173.87 ± 60.91 vs. 138.38 ± 57.38 min, *p* = 0.025), higher drainage volume (296.83 ± 282.97 vs. 150.83 ± 127.04 ml, *p* = 0.015), and longer postoperative hospital stay (7.97 ± 3.68 vs. 6.17 ± 1.63 days, *p* = 0.021). There was no mortality in all groups.

**Conclusions:**

LC or LCBDE is a safe and feasible procedure for experienced laparoscopic surgeons to perform on patients with previous upper abdominal surgery.

## Introduction

In recent years, laparoscopy's role in treating benign gallbladder and biliary tract diseases has been well confirmed (1). Laparoscopic cholecystectomy (LC) has the advantages of decreased pain, shorter convalescence, reduced operative stress, and limited inflammatory response. It has become the gold standard for treating benign gallbladder diseases (2, 3). Concerning common bile duct (CBD) stones, compared to other treatments, such as endoscopic retrograde cholangiopancreatography (ERCP), laparoscopic common bile duct exploration (LCBDE) has gained widespread acceptance because it preserves the function of the sphincter of Oddi, facilitates shorter hospital stay, and produces a comparable stone clearance rate (4). Previous upper abdominal surgery is an absolute contraindication to laparoscopy because of abdominal adhesion, which causes bowel or other abdominal structures to adhere to the abdominal wall (5). With the recent development of laparoscopic techniques and surgical skills, a previous upper abdominal surgery is considered only a relative contraindication (6). Many surgeons believe that laparoscopic procedures can also be performed as a standard treatment in patients with a history of abdominal operations. However, its safety and feasibility have not been thoroughly evaluated. We aimed to evaluate the clinical efficacy and safety of LC and LCBDE in patients with a history of right upper abdominal surgery or other upper abdominal surgery.

## Methods

### Patients and grouping

Of a total of 195 patients who met the inclusion criteria, the clinical data of 118 patients who underwent LC and 77 who underwent LCBDE at the Shengjing Hospital of China Medical University between September 2017 and September 2021 were analyzed retrospectively. Data about demographics and preoperative blood biochemical examinations were collected. The indications for LC include symptomatic gallstones, gallbladder polyps, and cholecystitis. The inclusion criteria for LCBDE include (1) CBD stones confirmed by abdominal ultrasound, computed tomography, or magnetic resonance cholangiopancreatography; (2) patients who were unsuitable for ERCP; (3) Patients who failed ERCP. We excluded patients suspected of gallbladder cancer, those with surgical contraindications, and those who underwent direct open surgery. We divided cases that met the inclusion criteria into the control and observation groups according to whether they had a history of upper abdominal surgery. The observation groups were divided into right upper abdominal surgery (groups A and C) and other upper abdominal surgery groups (groups B and D) according to the last operation site. The time since the last operation ranged from 2 months to 64 years. The areas of the last surgery are shown in Table 1. The Institutional Ethics committee of Shengjing Hospital approved the study protocol.

**Table 1:** Areas of previous upper abdominal surgery.

Group	Specific area of last surgery	Number
LC		83
Right upper abdominal surgery (group A)	Gallbladder (13)^a^ Liver (4)	17
Other upper abdominal surgery (group B)	Colon (7) Kidney (16) Stomach (14) Small intestine (11) Pancreas (3) Spleen (6) The entire abdominal cavity (9)	66
LCBDE		48
Right upper abdominal surgery (group C)	Gallbladder (16) CBD (13) Liver (1)	30
Other upper abdominal surgery (group D)	Colon (4) Kidney (2) Stomach (7) Small intestine (3) The entire abdominal cavity (2)	18

^a^ 13 patients underwent minimally invasive cholecystolithotomy using laparoscopy combined with choledochoscopy.

### Surgical technique

All patients underwent abdominal ultrasound, computed tomography, magnetic resonance cholangiopancreatography, or endoscopic ultrasonography to confirm the diagnosis preoperatively. The operations are performed by surgeons with substantial experience in laparoscopies (>500 cases of laparoscopic surgery have been successful).

#### LC

The surgery of the control group was performed using a standardized technique with three ports. The surgeon stood on the patient's left side and followed the principle of “critical view of safety” when using the ultrasonic scalpel to dissect the Calot's triangle. After confirming the cystic duct, common hepatic duct, and CBD, the cystic duct and cystic artery were clipped and transected. The gallbladder was dissected off the liver bed, put into the retrieval bag, and taken out. Hemostatic gauze and drainage tubes were applied during the operation according to the specific situation. In the observation group, an incision was made at the upper, lower, or both sides of the umbilicus according to the previous abdominal surgical incision site. After the pneumoperitoneum needle was successfully inserted into the abdominal cavity, the pneumoperitoneum machine was connected to insufflate carbon dioxide to maintain the intra-abdominal pressure at 12–14 mmHg. Furthermore, the abdominal wall was lifted, a 10-mm trocar was placed at the umbilical area, and the abdominal adhesions were explored through laparoscopy. Due to heavy adhesion under the incision, some patients were at a high risk of intestinal damage during puncture. We made a small incision at the first puncture port and cut off the abdominal wall layer by layer to establish a pneumoperitoneum under direct vision. The position of the other two trocars depended on the internal adhesions of the abdominal cavity, per the principle of triangular distribution. The rest of the surgical procedures were similar to those of the control group.

#### LCBDE

The location of the initial trocar and the establishment of the pneumoperitoneum were similar to those of LC. Under the guidance of the laparoscope, three more trocars were inserted: a 12-mm trocar was inserted at the subxiphoid as the main working port of the choledochoscope, and two 5-mm trocars were located at the middle of the right side and flank and used as auxiliary working ports. After routine exploration, an ultrasonic scalpel was used to dissect the adhesions attached to the previous incision, the right side of the liver, and around the gallbladder in the observation groups. The operation process of the control group was similar to that of the observation group; however, most patients did not require adhesion separation, and the surgical field of vision was excellent. The treatment of the gallbladder was similar to that of LC; however, the cystic duct was retained after being clipped for traction and cut off after the incision of the CBD was sutured. After the CBD was determined by needle aspiration, choledochotomy was performed with a 1–1.5 cm long vertical incision using electrocautery. Under the direct vision of a 5-mm fiber choledochoscope, the stones were removed by sterile saline irrigation, stone basket, or lithotripsy. After confirming that there were no residual stones through choledochoscopy, the T-tube was placed, and the CBD incision was sutured with vicryl 4–0 intermittently. Finally, an abdominal drainage tube was placed near the foramen of Winslow.

If a situation could not be handled under laparoscopy and other emergencies were suspected, it was converted to open abdominal surgery. The abdominal cavity drainage tube was removed when the drainage had stopped or became less than approximately 20 ml/day.

### Statistical analysis

Data analysis was performed using SPSS20 software (SPSS Inc., Chicago, IL, USA). The continuous variables were expressed as mean ± standard deviation, and the t-test was carried out. As appropriate, categorical data were compared using the Chi-square and Fisher exact tests. A *p*-value <0.05 was considered significant.

## Results

### Patient characteristics and preoperative observation index

Based on the demographic and biochemical test data (Table 2), there was no significant difference between the observation and control groups concerning gender, age, and preoperative white blood cell, alanine aminotransferase, aspartate aminotransferase, direct bilirubin, and total bilirubin levels.

**Table 2:** Patient characteristics and preoperative observation index.

	LC			LCBDE		
	Control	Group A	Group B	Control	Group C	Group D
Gender (M:F)	16:19	8:9	28:38	13:16	12:18	8:10
Age (years)	53.06 ± 13.19 (27–77)	55.29 ± 13.79 (20–77)	58.18 ± 13.64 (24–82)	56.38 ± 15.85 (35–73)	61.43 ± 7.14 (45–80)	61.89 ± 9.04 (50–86)
blood biochemical examinations						
WBC (*10^9^/L)	5.78 ± 1.82	5.63 ± 1.35	6.19 ± 2.11	6.27 ± 2.17	6.28 ± 2.52	5.37 ± 1.58
ALT (U/L)	50.06 ± 85.19	49.63 ± 58.84	25.48 ± 17.74	127.43 ± 179.71	100.23 ± 104.53	77.94 ± 99.52
AST (U/L)	25.00 ± 25.14	30.06 ± 21.36	25.39 ± 25.57	68.39 ± 92.95	67.57 ± 82.84	43.56 ± 35.33
BILD (µmol/L)	5.16 ± 4.38	5.43 ± 4.22	6.03 ± 4.70	18.35 ± 23.51	18.79 ± 26.68	11.49 ± 11.94
BILT (µmol/L)	13.13 ± 7.52	15.96 ± 9.56	15.86 ± 9.16	29.14 ± 28.49	30.01 ± 31.68	21.53 ± 16.40

All *p*-value >0.05; WBC: white blood cell, ALT: alanine aminotransferase, AST: aspartate aminotransferase, BILD: direct bilirubin, BILT: total bilirubin.

### Intraoperative observation indexes

Intraoperative observation indexes are shown in Table 3. The chief surgeon assessed the adhesions under direct laparoscopic vision. In the LC control group, 37.2% of the patients had no apparent adhesions, 45.7% had filmy adhesions, and only 17.1% had dense adhesions. The patients without apparent adhesion in groups A and B were fewer than those in the control group. The patients with dense adhesion reached 41.2% in group A and 45.5% in group B. Groups A and B had one case converted to open surgery due to extensive abdominal adhesions and difficulty separating adhesions under a laparoscope. The adhesions around the gallbladder in patients with a history of upper abdominal surgery were more serious, and laparoscopy was more complicated. The number of patients in group B who underwent electrocautery was significantly more than that in the control group (51.5 vs. 20.0%, *p* = 0.002). Although there were also more patients who underwent electrocautery in group A than in the control group, there was no statistical difference (29.4 vs. 20.0%, *p* = 0.45). There was also no significant difference between the observation and control groups in the usage rate of hemostatic gauze, intraoperative blood loss, and operation time.

**Table 3:** Comparison of operative results.

		LC			LCBDE		
		Control (*n* = 35)	Group A (*n* = 17)	Group B (*n* = 66)	Control (*n* = 29)	Group C (*n* = 30)	Group D (*n* = 18)
Adhesion *N* (%)	No adhesions	13 (37.2)	5 (29.4)	17 (25.7)	7 (24.1)	3 (10.0)	1 (5.6)
	Filmy Ddhesions	16 (45.7)	5 (29.4)	19 (28.8)	17 (58.6)	5 (16.7)	7 (38.9)
	dense adhesions	6 (17.1)	7 (41.2)	30 (45.5)	5 (17.3)	22 (73.3)	10 (55.5)
Estimated blood loss (ml)		15.14 ± 22.80	13.13 ± 6.80	18.77 ± 16.65	18.97 ± 13.12	41.33 ± 50.84*	66.11 ± 87.46*
Hemostasis *n* (%)	Electrocautery	7 (20.0)	5 (29.4)	34 (51.5)*	29 (100)	30 (100)	18 (100)
	Hemostatic gauze	9 (25.7)	5 (29.4)	19 (28.8)	14 (48.3)	25 (83.3)*	12 (66.7)
Operation time (min)		63.74 ± 24.00	75.06 ± 33.90	70.00 ± 30.00	138.38 ± 57.38	173.87 ± 60.91*	128.61 ± 48.95

* *p*-value <0.05.

In the patients undergoing LCBDE, those with no apparent adhesions accounted for 24.1% in the control group, 10.0% in group C, and 5.6% in the group D; the dense adhesions accounted for 17.3% in the control group, 73.3% in the group C, and 55.5% in the group D. The estimated intraoperative blood loss of the observation groups was significantly higher (group C, 41.33 ± 50.84 ml, *p* = 0.026; group D, 66.11 ± 87.46 ml, *p* = 0.036). Electrocautery was routinely used in LCBDE; however, the usage rate of hemostatic gauze was significantly higher in group C than in the control group (83.3 vs. 48.3%, *p* = 0.004). The operation time was not significantly different between the control group and group D but longer in group C (173.87 ± 60.91 vs. 138.38 ± 57.38 min, *p* = 0.025).

### Postoperative observation indexes

The biochemical blood examinations were performed on the first day after the operation in each group. The laboratory test results, such as white blood cell, alanine aminotransferase, aspartate aminotransferase, direct bilirubin, and total bilirubin levels, were not statistically different between the groups (Table 4).

**Table 4:** Comparison of postoperative laboratory tests.

	LC			LCBDE		
	Control	Group A	Group B	Control	Group C	Group D
WBC (*10^9^/L)	9.29 ± 2.32	9.58 ± 3.37	9.68 ± 4.02	12.69 ± 4.29	12.70 ± 4.03	11.32 ± 3.12
ALT (U/L)	62.83 ± 76.04	57.82 ± 60.71	45.36 ± 44.37	49.12 ± 32.84	66.27 ± 28.84	49.06 ± 32.78
AST (U/L)	46.09 ± 41.63	38.94 ± 28.30	42.95 ± 39.78	46.03 ± 23.35	49.13 ± 26.91	44.28 ± 17.70
BILD (µmol/L)	8.46 ± 7.93	6.46 ± 2.86	8.71 ± 11.07	11.14 ± 7.03	15.34 ± 14.06	12.52 ± 8.53
BILT (µmol/L)	20.77 ± 11.71	18.66 ± 7.68	21.37 ± 16.39	21.73 ± 8.96	27.26 ± 17.38	24.40 ± 13.82

All *p*-value >0.05.

Among the patients undergoing LC, 14.3% of patients in the control group received a drainage tube, with an average drainage volume of 26.57 ± 69.77 ml; 29.4% of patients in group A (*p* = 0.194), with an average drainage volume of 50.06 ± 83.66 ml (*p* = 0.291); 25.8% of patients in group B (*p* = 0.184), with an average drainage volume of 60.29 ± 123.78 ml (*p* = 0.140). There was no statistical difference between the groups. The postoperative hospital stay and hospitalization costs were also not significantly different between the LC groups (Table 5). However, a patient in group B, who underwent subtotal gastrectomy 17 years ago, had intra-abdominal hemorrhage after LC and underwent laparotomy to stop bleeding. No related complications occurred in the control group and group A.

**Table 5:** Postoperative observation indexes and complications.

	LC			LCBDE		
	Control	Group A	Group B	Control	Group C	Group D
Drainage volume (ml)	26.57 ± 69.77	50.06 ± 83.66	60.29 ± 123.78	150.83 ± 127.04	296.83 ± 282.97*	176.11 ± 174.76
Postoperative hospital stay (days)	4.09 ± 1.04	4.00 ± 1.46	4.48 ± 1.46	6.17 ± 1.63	7.97 ± 3.68*	6.61 ± 3.07
Hospitalization costs (RMB)	23,636.85 ± 7,063.29	26,932.54 ± 11,898.31	26,160.39 ± 6,972.70	41,108.36 ± 11,130.72	48,178.34 ± 22,038.57	45,398.03 ± 11,546.99
Postoperative complication	0	0	1 (1.5%)	0	2 (6.7%)	0

* *p*-value <0.05.

In the patients undergoing LCBDE, the average drainage volume of group C was significantly higher than that of the control group (296.83 ± 282.97 vs. 150.83 ± 127.04, *p* = 0.014). The postoperative hospital stay in group C was also slightly longer (7.97 ± 3.68 vs. 6.17 ± 1.63 days, *p* = 0.021). There was no significant difference in hospitalization expenses among the groups. In group C, one patient with recurrent fever due to pelvic abscess was treated by abscess puncture; another patient with abdominal effusion after LCBDE operation received abdominal puncture treatment. No related complications occurred in the remaining groups.

In addition, based on the Clavien–Dindo classification system, we compared multiple groups of complications of each group and its corresponding control group (Table 6). We found that among groups A (*p* = 0.598), B (*p* = 0.476), C (*p* = 0.724), and D (*p* = 0.791), there was no significant difference between LC or LCBDE surgery and its control group, respectively.

**Table 6:** The complication grades according to the Clavien–Dindo classification system.

Grade according to the Clavien–Dindo classification system (%)	LC			LCBDE		
	Control (*n* = 35)	Group A (*n* = 17)	Group B (*n* = 66)	Control (*n* = 29)	Group C (*n* = 30)	Group D (*n* = 18)
I	34 (97.14)	16 (94.12)	62 (93.94)	25 (86.21)	25 (83.33)	16 (88.89)
II	1 (2.86)	1 (5.88)	3 (4.54)	4 (13.79)	4 (13.33)	2 (11.11)
III	0	0	1 (1.52)	0	1 (3.34)	0
IV	0	0	0	0	0	0

All *p*-value >0.05.

## Discussion

Compared with open cholecystectomy, LC can reduce postoperative pain, allow earlier oral intake, shorten hospital stays, promote earlier return to normal activities, and provide better cosmetic results. With the development of laparoscopic technology and equipment, LC has become the gold standard for treating benign gallbladder diseases (7, 8). There are currently more options for treating CBD stones. ERCP and LCBDE have become the two primary minimally invasive treatment options in clinical practice (9). The advantage of LCBDE is that only a single-stage approach is required for patients with CBD stones and gallstones while retaining the function of the sphincter of Oddi and preventing ERCP-related complications. Therefore, when ERCP is difficult or fails to remove stones, LCBDE is the best alternative treatment (10).

However, in the early period of laparoscopic surgery, previous abdominal surgery is widely regarded as an absolute contraindication to laparoscopy. The main reason is the adhesions formed during repair and healing after abdominal surgery. In this study, we evaluated the adhesion of all cases, and the results showed that the adhesion of the operation area in the observation groups was significantly more severe than that in the control group, particularly in patients with a history of right upper abdominal surgery. These adhesions have resulted in many problems in laparoscopy as follows: (1) it is challenging to establish pneumoperitoneum and access to the abdomen, and it is easy to injure the organs that adhered after the last surgical incision; (2) the exposed surgical vision is inadequate, and the anatomical structure is unclear; (3) the possibility of secondary injuries, such as bile leakage and intestinal fistula while separating adhesion; (4) higher conversion rate to laparotomy (4, 11). With the recent development of laparoscopic techniques and surgical skills, the history of upper abdominal surgery is no longer an absolute contraindication to laparoscopy. LC can be safely used in patients with previous upper abdominal surgery (12, 13). Li et al. suggested that LCBDE should be recommended for patients with a history of cholecystectomy, few previous operations (<2 times), or a history of laparoscopy (14). In this study, 131 patients with a history of various upper abdominal surgery were enrolled, of which 83 underwent LC and 48 cases underwent LCBDE. Our results showed that in LC, there was no significant difference between the observation and control groups in terms of laboratory examination, estimated blood loss, operation time, drainage volume, and postoperative hospital stay. In LCBDE, compared with the control group, patients with a history of right upper abdominal surgery had slightly higher estimated blood loss, longer operation time, more drainage volume, and more extended postoperative hospital stay; the patients with a history of other upper abdominal surgery only had higher estimated blood loss than the control group. It can preliminarily indicate that the history of right upper abdominal surgery has a more significant impact on LCBDE than other upper abdominal surgery. The difference in results between the two operations may be because LCBDE is a more complicated procedure than LC, and adhesions significantly impact LCBDE. Based on our research, we believe that the history of upper abdominal surgery is a relative contraindication to laparoscopic surgery.

We agree with the conclusions of some studies that abdominal adhesions directly affect the establishment of pneumoperitoneum, the insertion of trocars, and subsequent laparoscopic operations, increasing difficulty in operation, rate of intraoperative injuries, and postoperative complications. The occurrence of such cases is closely related to the skill level and experience of laparoscopy (15, 16). Therefore, we summarized some key points from the operations. Firstly, for patients with a history of upper abdominal surgery, the successful establishment of pneumoperitoneum is the key to successful LC or LCBDE. Preoperatively, it is necessary to inquire about the last operation in detail, evaluate the degree and area of abdominal adhesion in advance, and choose the first puncture point carefully. Karayiannakis et al. proposed that ultrasound detection is an effective method for preoperatively diagnosing adhesions under the umbilicus and abdominal wall incisions (13). We generally chose the umbilicus as the first puncture point and used the Kocher clamp to lift the abdominal wall on both sides of the umbilicus to increase the space and avoid puncturing the bowel loop. If it is estimated that the adhesions under the abdominal wall around the umbilicus are severe and unsuitable for a direct puncture, the open method (Hasson technique) can be used to establish pneumoperitoneum. After the pneumoperitoneum is established, the rest of the trocars are placed under direct vision. The location and number of trocar ports should be determined according to the internal adhesion of the abdominal cavity to meet the surgical requirements. Sometimes adding an operating hole can significantly improve operating conditions. In this study, two patients in group B completed LC under four holes and received excellent results, making the operation better and faster.

During the operation, it is not necessary to lyse all the adhesions seen under the laparoscope, only those adhesions that truly interfere with visualizing the area of interest or would prevent the placement of subsequent cannulas under vision should be lysed (6). Excessive separation will only prolong the operation time and increase the risk of organ injury and bleeding. We followed the above principle to eliminate adhesion and found that patients with a history of other upper abdominal surgery have shorter operation times and less drainage than those with a history of right upper abdominal surgery. To avoid thermal damage, we recommend using an ultrasonic scalpel instead of electrocautery to separate the adhesion. Blunt separation with the head of the aspirator helps to identify critical anatomical structures. In case of inaccessible tissue spaces with dense adhesion, it should be converted to laparotomy promptly to prevent unnecessary damage. In the LC observation groups, two patients were switched to laparotomy due to extensive dense adhesions in the abdominal cavity; they recovered well after the operation. Therefore, we should correctly understand the conversion to laparotomy during laparoscopy. It should not be seen as a technical failure but rather as a safer way to avoid complications and accomplish the same therapeutic goal (16, 17). Being able to grasp the timing and take the initiative to convert to laparotomy before the occurrence of serious complications is a sign of a mature laparoscopic surgeon.

The wound left by adhesion separation may bleed due to repeated rubbing of instruments during an operation. Suspicious wounds should be checked multiple times to ensure the safe completion of the operation. Generally, the surgeon will apply hemostatic gauze or sponge according to the exudation, which helps prevent bleeding. For obvious bleeding, we can also use electrocautery to stop bleeding. The main complications of LC and LCBDE are bleeding and bile duct injury. The postoperative complications are mainly bile leakage, bleeding, and liver abscess (18, 19). Bile leakage or bleeding may occur after an operation in the case of large surgical wounds and excessive bleeding. A suction drain tube should be routinely placed near the foramen of Winslow for drainage. If necessary, another drain tube can be placed under the liver. Indwelling drainage plays a role in local drainage, which facilitates recovery. More importantly, it can help us to observe bleeding, biliary fistula, and intestinal fistula that were not discovered promptly during the operation for rapid and proper treatment. One patient in group B had postoperative abdominal bleeding, and one in group C had postoperative abdominal effusion. They were all discovered promptly through indwelling drainage and treated accordingly. The drainage tube is unnecessary for those who do not have obvious exudation and bleeding. In LCBDE, the choice of primary closure and T-tube drainage is also a widespread concern. T-tube drainage can reduce the risk of bile leakage and provide a channel for postoperative identification and removal of residual stones. However, many studies suggest that the primary closure had significantly better outcomes when compared to T-tube drainage, and it can be routinely performed as an alternative to T-tube drainage (20–22).

In summary, it is still safe and feasible for experienced laparoscopic surgeons to perform LC or LCBDE on patients with a history of upper abdominal surgery. Our primary results show that there is no significant difference in the outcomes of LC between patients with and without a history of upper abdominal surgery. For patients who underwent LCBDE, those with a history of right upper abdominal surgery have a longer operation time, more estimated blood loss, more drainage volume, and a longer postoperative hospital stay than other groups. These differences may be related to surgical experience, degree of adhesion, and anatomical variation.

## Data Availability

The original contributions presented in the study are included in the article/Supplementary Material, further inquiries can be directed to the corresponding author/s.
